# Can we stop AIDS with antiretroviral-based treatment as prevention?

**DOI:** 10.9745/GHSP-D-12-00053

**Published:** 2013-03-21

**Authors:** Edward J Mills, Jean B Nachega, Nathan Ford

**Affiliations:** a Stanford Prevention Research Center, Stanford University, Stanford, CA, USA; b Johns Hopkins Bloomberg School of Public Health, Baltimore, MD, USA; c Stellenbosch University, Centre for Infectious Diseases, Cape Town, South Africa; d Médecins Sans Frontières, Geneva, Switzerland

## Abstract

Challenges to scaling up treatment as prevention (TasP) of HIV transmission are considerable in the developing-world context and include accessing at-risk populations, human resource shortages, adherence and retention in care, access to newer treatments, measurement of treatment effects, and long-term sustainable funding. Optimism about ending AIDS needs to be tempered by the realities of the logistic challenges of strengthening health systems in countries most affected and by balancing TasP with overall combination prevention approaches.

The 2011 results of the HIV Prevention Trials Network 052 randomized clinical trial (RCT)^1^ evaluating antiretroviral treatment as prevention (TasP) of HIV transmission heralded a new era of HIV/AIDS control as the debate about prioritizing treatment or prevention comes to an end.^2^ For many years, the best hope for ending the HIV epidemic was thought to lie in the development of an effective vaccine. But for now, the most effective preventive interventions will come from tools we already have, including antiretroviral therapy (ART), pre-exposure prophylaxis (PrEP) with ART, male circumcision, and condoms.

There is broad consensus that prevention strategies need to involve a combination of proven prevention interventions.^3^ There is also strong advocacy that TasP should be the backbone of population-based prevention.^3-4^ With considerable enthusiasm, the international research community has produced mathematical models of TasP to postulate the end of AIDS, an AIDS-free generation, and a cost-effective strategy that saves billions of investments in the future.^5-6^ Yet such widespread enthusiasm needs to be tempered with programmatic realities. In the era of global economic uncertainty, we need to overcome a number of specific challenges to realize any population-wide benefits of TasP.

## EVIDENCE FOR TASP

Mathematical models may be useful for raising policy implications, but they are highly susceptible to the assumptions that inform them. The heterogeneity of estimates of TasP benefits weakens their inferences severely.^6^

As early as 1991, mathematical projections highlighted the potential for TasP using only the antiretroviral drug zidovudine.^7^ Since then, incrementally stronger evidence from cohort evaluations have indicated that transmission risks are associated with specific viral load thresholds.^8^ It was not until 2011 that results of the HPTN 052 RCT provided randomized evidence of a large preventive benefit associated with early provision of antiretroviral treatment. Early treatment resulted in a 96% reduction in the number of HIV transmissions compared with delayed treatment (95% confidence interval [CI], 73% to 99%).^1^

The trial was conducted among serodiscordant couples (one partner had HIV infection and the other did not) in a well-monitored RCT environment. However, the randomized portion of the trial was stopped early due to treatment benefits,^9^ but it was based on comparatively few events^10^—both issues that may bias a trial in favor of inflated treatment effects.^11^ In the most recent evaluation of TasP among discordant couples in China, treatment of the index partner conferred only a 26% reduction in transmission to the non-infected partner compared with no treatment (95% CI, 16% to 35%).^12^ These findings indicate that the large treatment effects observed in the HPTN 052 RCT are unlikely to be fulfilled in real-world, non-trial environments.

## CHALLENGES

In light of this uncertain evidence, there are a number of important challenges to scaling up TasP, categorized into 6 distinct areas:

Early provision of ART reduces transmission of HIV infection—by as much as 96% in randomized trials. But benefits in the real world are probably markedly lower.

1. Prioritization of patient and population groups
2. Human resources and health systems
3. Acceptance, adherence, and retention of patients
4. Improved access to more effective therapies
5. Tools to measure the effect of TasP
6. Financial resources to cover new costs

Many of these issues were put forward a decade ago as challenges to scaling up ART in under-resourced settings.^13^

### Priority Populations

The greatest public health benefits in targeted prevention come from stopping transmissions among those who are most likely to infect others. One approach to maximizing the benefits of TasP should be to address specific groups of individuals with HIV infection, such as sex workers, injecting drug users, men who have sex with men (MSM), and individuals with multiple sex partners. There is an expectation that targeting screening and treatment (test and treat) to specific groups of people who are at high risk of transmitting the virus—and thus reducing their viremia—may result in the greatest reduction in new infections, although this has yet to be evaluated in any research setting.^14^ Moreover, these groups are precisely the groups that, in many contexts, have been the hardest to reach for HIV testing and treatment programs because of stigma and discrimination; in many African countries where HIV burden is highest, injecting drug users, sex workers, and MSMs are criminalized.

While there is clearly a need to better engage these key affected populations in TasP programs, the decision to prioritize screening and treatment based on certain risk behaviors rather than on medical need continues to be a subject of debate.^2^ An alternative approach would be to increase treatment coverage for people who are clinically eligible by scaling up HIV counseling and testing (HCT) campaigns, linked with rapid eligibility assessment through, for example, point-of-care CD4 testing.^15^ This population may not have diverse links within transmission networks. However, many would argue that this group of people should be prioritized over healthier people with HIV infection because they represent a population that the clinical community has failed to adequately serve and because providing treatment to patients with high viremia and low CD4 counts may still have considerable preventive benefits.^16^

Other strategies to engage infected individuals early besides point-of-care testing include self-testing and home-based testing visits. A recent evaluation of house-to-house testing by health workers in Kenya found high levels of acceptance and that people accepting home testing had a median CD4 count of 323 cells/µL compared with a median CD4 count of 217 among those visiting voluntary testing centers.^17^

### Human Resources

With increasing numbers of individuals screened and treated, health systems will need larger numbers of trained health care workers capable of testing, initiating treatment, monitoring adherence and retention, and engaging patients in long-term social support groups. In countries with HIV burden, health service constraints to expanding ART access are considerable. For example, in settings such as Swaziland (HIV prevalence 25%) and Malawi (prevalence 11%), there are fewer than 0.05 physicians per 1,000 people and fewer than 0.2 nurses per 1,000 people.^18^

The simplification of screening, treatment, and monitoring of patients has allowed for task shifting from doctors to less specialized health staff. Despite numerous observational studies and several randomized trials validating this approach, task shifting for HIV treatment and care is still not universally accepted.^19^

Increasing numbers of patients engaged in treatment will also require that health systems adapt from facility-based care to community-based care and delivery. Several important examples of this model exist in Kenya, Mozambique, and Uganda.^20-22^ If more patients begin treatment at higher CD4 counts—before they become sick—then there may be no particular reason for them to routinely visit the central health system if quality care can be delivered to their local communities.

TasP provides an additional compelling argument for improving access to viral load monitoring as CD4 is a weak surrogate marker of viral suppression. Currently, due to costs, viral load monitoring is rarely provided in the lowest-resource settings. Advocates of viral load monitoring over CD4 monitoring argue that viremia is a better predictor of treatment failure than CD4 monitoring and that a renewed emphasis on decreasing costs of care should focus on making viral monitoring available in resource-limited settings.^23^

Viral load monitoring may be a better predictor of ART failure than CD4 monitoring.

### Acceptance, Adherence, and Retention

Linking patients with a positive HIV test to effective and uninterrupted ART represents an important challenge. Major attrition of patients occurs between each stage of the leaky treatment cascade—from diagnosis and assessment of ART readiness to receipt of initial ART and long-term retention in care (Figure).^24^

**FIGURE.**
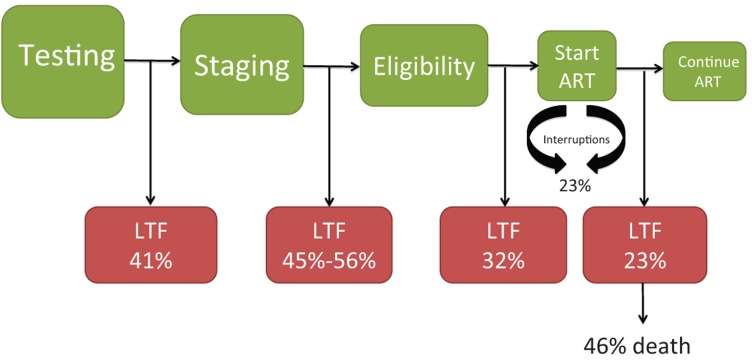
The Leaky HIV Treatment Cascade The cascade of HIV care proceeds from testing and clinical staging to ART eligibility, receipt of ART, and successful, uninterrupted treatment. Each step opens the possibility to losing patients to follow-up, which has been documented by a number of studies.^25-27^ Abbreviations: ART, antiretroviral therapy; LTF, loss to follow-up.

Just because patients have been prescribed ART does not mean they are willing to begin lifelong treatment. A recent study from Kenya found that almost 40% of serodiscordant couples were unwilling to use early treatment for its preventive effects.^28^

Systematic reviews show that, on average, adherence among people with HIV infection that do start ART is below 80%,^29^ and around a quarter of patients interrupt treatment for a median of 150 days.^30^ In Uganda, a study found that 11% of patients took unstructured treatment interruptions for over a year and patients with higher CD4 counts were more likely to discontinue care.^31^

Reducing viremia effectively requires adequate and sustained adherence, but approaches to improve adherence remain poorly defined. Community-based organizations have used social groups, involved partners, and sent mobile phone text messages in efforts to retain patients along the cascade of care.^25^ Despite dozens of proposed adherence-support interventions, only counseling and mobile phone text messaging have demonstrated their utility in large randomized trials.^32^

Treating greater numbers of asymptomatic patients will require renewed efforts to improve adherence, including identifying adherence-support models for people who have never experienced an illness episode.

Early treatment of HIV patients who may not have symptoms yet requires enhanced efforts to improve adherence.

### Improved Access to More Effective Therapies

Although there have been recommendations to shift away from using drugs associated with severe adverse events, such as stavudine, toward better-tolerated tenofovir-based formulations, stavudine is still commonly prescribed in many resource-limited settings.^33^ Earlier initiation of ART is a balance of risks. In Western countries, a move toward earlier initiation was contemplated only once less toxic drugs became available. Likewise, a move toward widespread provision of antiretrovirals to asymptomatic patients in other countries will require improving the availability of better-tolerated regimens.^34^

### Tools to Measure the Effect of TasP

Measuring whether TasP has a marked decrease on new infections in communities represents an important challenge to scale up.^35^ Only incidence monitoring can determine whether rates of infection have gone up or down. Monitoring HIV prevalence data from cross-sectional surveys may be misleading because other unmeasured interventions, such as prevention messaging, circumcision, death, and the natural course of the disease, may affect prevalence rates. For example, Uganda experienced a marked decrease in HIV prevalence estimates between 1995 and 2000 in the absence of ART, mostly due to the high number of AIDS-related deaths and to successful HIV-prevention media campaigns.

Incidence monitoring is challenging in many settings in Africa because few incidence cohorts exist. Also, many populations at high risk of infection, such as migrant workers, have high rates of mobility and may not be captured in incidence surveys.

Additionally, determining the specific preventive contribution of treatment will be difficult in an environment where a range of positive prevention interventions, such as condom and clean-needle use, male circumcision, and decreasing the number of partners, are used by different populations to different extents. In 2 pragmatic, controlled cohorts of serodiscordant couples (in China and Uganda) where the index partner in the intervention group received ART, there were no differences in the rates of infection between groups.^36-37^ While concerns about study quality afflict the China study,^38^ in the Uganda study, the lack of reduced HIV transmission risk occurred despite high levels of viral suppression in the ART group.^37^

### Costs

Implementing treatment as a prevention strategy will have financial costs associated with it in terms of drugs, human resources, laboratory monitoring, and evaluation. In the current global economic climate, where this money will come from is a mystery.

In more developed settings, such as British Columbia, Canada, financial resources for TasP are obtained by moving resources from a previous allocation to the seek-and-treat program.^39^ In settings that rely on development assistance initiatives, such as the U.S. President's Emergency Plan for AIDS Relief (PEPFAR) or the Global Fund to Fight AIDS, Tuberculosis and Malaria, new financial resources are unlikely.

Most organizations in Africa have a set allocation of patients who are permitted to begin treatment within defined funding periods, and they are not permitted to initiate ART for patients who do not meet their organization's initiation criteria. Engaging a large number of new patients with higher CD4 status than current eligibility criteria will require permission from funders. Yet neither PEPFAR nor the Global Fund has announced the allocation of significant new funding for TasP.

Whether TasP is a cost-effective strategy compared with other preventive approaches in HIV-endemic settings has been controversial. A model evaluating the cost-effectiveness of TasP compared with medical male circumcision found that a focus on circumcision had greater cost-effectiveness than TasP alone.^40^ Clear evidence on the preventive effects of male circumcision^41^ has been available since 2005, but the practice has not been widely implemented, demonstrating that evidence alone does not drive funding or policy.

## CONCLUSIONS

Important programmatic challenges have hindered scale up of ART for the purpose of treatment alone.^13^ Enthusiasm about scaling up treatment for preventive effects needs to be tempered by the reality that these programmatic challenges may be difficult to overcome. Overly optimistic public health messages about the preventive benefits of treatment have resulted in misleading communication that effective treatment will reduce the need for other preventive techniques, such as condoms.^42^ The extent to which this results in modifications in risky behavior should be of paramount concern to those involved in public health messaging.^43^

While treatment as prevention holds promise, scale-up strategies need to be mindful of programmatic challenges.

TasP has given much-needed impetus to the HIV-prevention and treatment agenda at a time when political support was waning. It has also focused attention on the health system challenges to enrolling and retaining more people with HIV infection, earlier in care.

Other interventions, such as PrEP, prevention of mother-to-child transmission (PMTCT), condom and clean-needle use, and medical male circumcision, are also important strategies to prevent infections. All these interventions face challenges in terms of timely uptake by individuals at risk, health service capacity, and, in the case of PrEP and PMTCT, adherence to treatment.

The appropriate strategic combination of these different biomedical prevention interventions will differ according to the epidemiologic, economic, and cultural realities of different settings.^44^ Approaches to defining the best combination prevention mix for particular settings is perhaps one of the most important implementation research questions in HIV prevention today.
